# Clinical Stages in Patients with Primary and Subsequent Cancers Based on the Czech Cancer Registry 1976–2005

**DOI:** 10.1155/2013/829486

**Published:** 2013-07-07

**Authors:** Edvard Geryk, Radim Štampach, Petr Dítě, Jiří Kozel, Teodor Horváth, Petr Kubíček

**Affiliations:** ^1^Department of Science and Research, University Hospital Brno, Jihlavska 20, 625 00 Brno, Czech Republic; ^2^Laboratory on Geoinformatics and Cartography, Department of Geography, Faculty of Science, Masaryk University, Kotlarska 2, 611 37 Brno, Czech Republic; ^3^Department of Internal Medicine, University Hospital Brno, Pekarska 53, 602 00 Brno, Czech Republic; ^4^Department of Surgery, University Hospital Brno, Jihlavska 20, 625 00 Brno, Czech Republic

## Abstract

Of 1,486,984 new cancers registered in the Czech Cancer Registry in 1976-2005, 290,312 (19.5%) were multiple malignant neoplasms (MMNs), of which there were 65,292 primary and 89,796 subsequent cases in men and 59,970 primary and 75,254 subsequent cases in women. The duplicities were higher in women, and the triplicities and others (3–6 MMNs) were higher in men. The most frequent diagnoses were the primary cancers of skin, gastrointestinal and urinary tract, male genital organs, respiratory tract in men, and cancers of skin, breast, female genital organs, and gastrointestinal tract in women. The analysis of the early and advanced clinical stages shows that the number of subsequent advanced stages increased after primary advanced stages. Their time-age-space distributions visualized maps of MMNs in 14 Czech regions. These results support the improvement of algorithms of dispensary care for the early detection of the subsequent neoplasms.

## 1. Introduction

The health status of the Czech population can be seen as very vulnerable, mainly because of the high risk of cancer especially in younger age. This fact is confirmed by data in Globocan 2008 [1]. Cancer diagnoses are registered since 1959. The IARC criteria are used were for their notificationed since May 1976 by criteria of the IARC. The annual surveys of Health Information and Statistics of the Czech Republic [2] confirmed the continued trend of cancer occurrence, observed in the Czech areas from 1905 [3] and continuously described from 1933 [4]. The cancer incidence increased from 24,471 (254.4/100,000) in 1959 over 35,407 (347.5/100,000) in 1977 to 78,846 (751.5/100,000) in 2009 [2]. In view of new diagnostic and treatment modalities, the prevalence of cancers (ICD-10: C00-97, D00-09) increased from 174,311 (1,682.2/100,000) in 1989 to 461,545 (4,510/100,000) in 2005. Under the conditions of continuous diagnostics, treatment, medical surveillance, and cancer evidence, the survivors can reach in 2015 nearly 317,000 cases in men (of which 33.2% in age 35–64 years) and 434,000 in women (of which 42.6% in age 35–64 years) [5]. The differences between the numbers of cases and numbers of persons indicated the multiple malignant neoplasms (MMNs). After the preliminary report of their trend [6], this paper is another contribution to this issue.

## 2. Methods

The data of MMNs were based on the number of cancers reported to the Czech Cancer Registry between May 1976 and December 2005 and were verified and anonymised up to October 17, 2007. The percentage of multiple cancers of all diagnoses (ICD-10: C00-97, D00-09, D37-48, i.e. including the skin cancer) in males and females was compared with the number of newly diagnosed cases in the same codes. The primary and subsequent cancers were prepared in contingency tables and analysed by age, time and space distribution, and clinical stages of early (ST I, II), advanced (ST III, IV), and unknown cases. Their input data correspond with the classification of diseases at the time of diagnosis and with the level of cancer evidence in 14 Czech regions.

## 3. Results

A total of 1,486,984 new malignant neoplasms (50.9% men, 49.1% women) were registered in the Czech Cancer Registry between 1976 and 2005. This number includes 1,430,458 (96.2%) cancers (C00-97), 42,630 (2.9%) neoplasms in situ (D00-09), and 13,896 (0.9%) neoplasms of uncertain behaviour (D37-48). Of all newly registered cancers, there were notified 290,312 (19.5%) MMNs. In men were diagnosed 65,292 primary and 89,796 subsequent cancers and in women 59,970 primary and 75,254 subsequent cancers. A total of 84% duplicities were higher in women than 79.6% in men, while triplicities and others (3–6 MMNs) were in 19.1% cases of men and 15.4% of women. In men, there were the most frequent primary cancers of skin 46%, gastrointestinal 13.5% and urinary tract 9.6%, male genital organs 8.1% and respiratory tract 7.7%; in women, there were cancers of skin 39.4%, breast 17.3%, female genital organs 14.7%, and gastrointestinal tract 9.8%. The most frequent MMNs were 53,616 primary and 70,119 subsequent cancers of skin as a warning sign for the risk of following neoplasms. The most frequent subsequent diagnoses following primary cases were 26,790 cancers of gastrointestinal tract (54.6% men, 45.4%), 12,801 of respiratory tract (76.9% men, 23.1% women), 10,704 of urinary tract (66.6% men, 33.4% women), 9,394 of breast (0.9% men, 99.1% women), 5,284 of lymphoid and haematopoietic tissue (54.3% men, 45.7% women), and 6,804 of male and 9,309 of female genital organs (Table 1).

**Figure 1:**
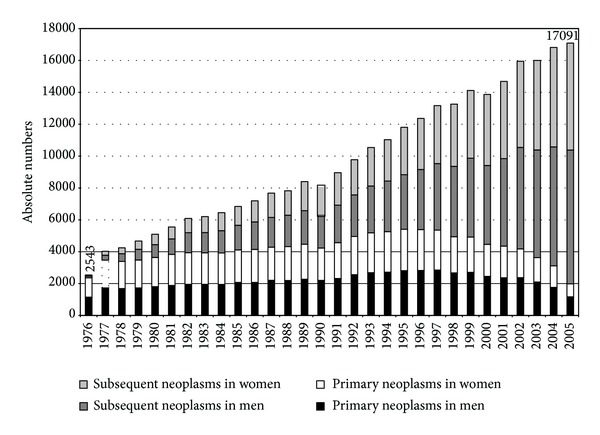
Trend of multiple malignant neoplasms in the Czech Republic 1976–2005.

The yearly number of MMNs increased from 2,543 cases in 1976 to 17,091 in 2005, of which the primary cancers increased from 2,365 in 1976 to 5,411 in 1995 and then decreased to 1,983 in 2005, while the subsequent cancers increased from 178 in 1976 to 15,108 in 2005 (Figure 1). The predominance of unknown stages in both sexes over other stages lasted until 1994; from the next years their number decreased especially in the early stages of primary cancers. The percentage occurrence of stage IV was permanently higher in men than in women. 

Let us see the situation in age groups. The values of the primary cancers in age group up to 49 years are 8.3% cases in men and 15.7% in women, in group 50–69 years 52.4% in men and 47.8% in women, in group 70–79 years 31.3% in men and 26.7% in women, and in group over 80 years 8% in men and 9.8% in women. After the exclusion of unknown stages, the numbers of early stages were higher in men in group of 70–79 years, in women in group up to 49 years, and in group over 80 years. The numbers of advanced stages were higher in men in group of 50–79 years.

Of the total 65,292 primary cancers in men, were diagnosed 24,263 (37.1%) cases of early stages (of which 28.2% ST I) and 6,051 (9.3%) cases of advanced stages (of which 4.2% ST IV); the unknown stages featured 34,978 (53.6%) cases. Of the total 59,970 primary cancers in women were diagnosed 27,922 (46.6%) cases of early stages (of which 32.6% ST I), 5,707 (9.5%) cases of advanced stages (of which 3.2% ST IV); the unknown stages featured 26,341 (43.9%) cases. The specific position presents 22,607 subsequent cancers of advanced stages (ST III, IV), of which 6,172 cases (i.e. 12.4% of 49,717) followed primary ST I, 2,865 cases (i.e. 17.3% of 16,533) followed primary ST II, 1,905 cases (i.e. 24.1% of 7,890) followed primary ST III, 1,355 cases (i.e. 27.2% of 4,976) followed primary ST IV, and 10,310 cases (i.e. 12% of 85,934) followed primary unknown stages (Table 2). The number of subsequent advanced stages increased with the advanced stage of primary disease. 

The geographical distribution of MMNs by stages during 1976–2005 shows relevant maps (Figures 3, 4, 5, 6, 7, 8, 9, 10, and 11). The most frequent values of primary-subsequent cancers of 14 regions reached the population of Northern Moravia—primary 12.7% and subsequent 13.7%, Prague—primary 11.8% and subsequent 11.8%, and Southern Moravia—primary 11.8% and subsequent 11.7%. The distribution of advanced stages presents two percentage values. 

**Figure 2:**
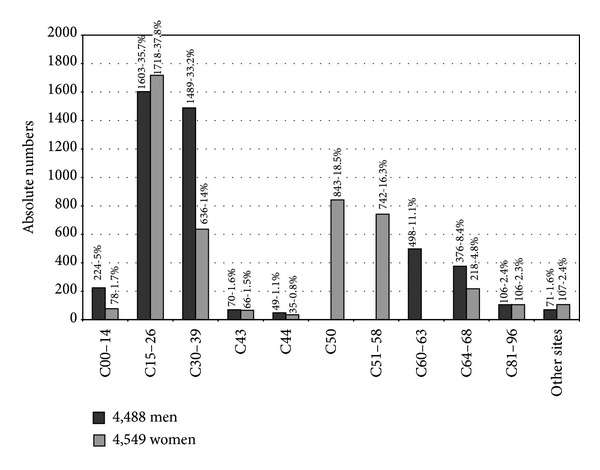
Subsequent diagnoses of advanced stages following primary neoplasms of early stages.

**Figure 3:**
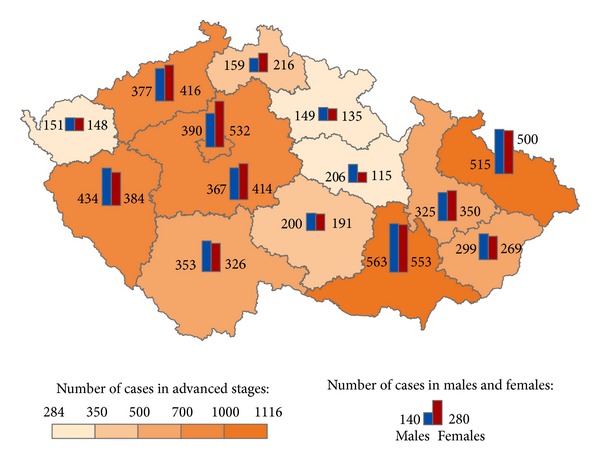
Advanced clinical stages of subsequent neoplasms after early clinical stages of primary neoplasms in regions of the Czech Republic in 4,488 males and 4,549 females in 1976–2005.

**Figure 4:**
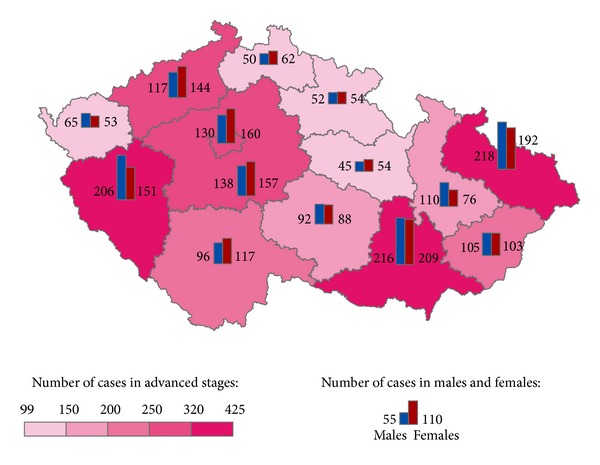
Advanced clinical stages of subsequent neoplasms after advanced clinical stages of primary neoplasms in regions of the Czech Republic in 1,640 males and 1,620 females in 1976–2005.

**Figure 5:**
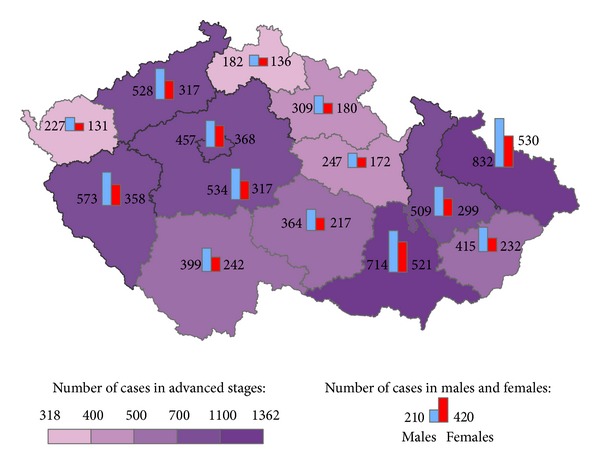
Advanced clinical stages of subsequent neoplasms after unknown clinical stages of primary neoplasms in regions of the Czech Republic in 6,290 males and 4,020 females in 1976–2005.

**Figure 6:**
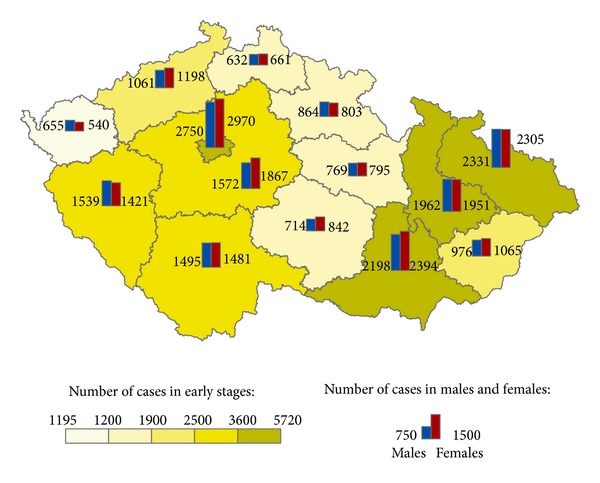
Early clinical stages of subsequent neoplasms after early clinical stages of primary neoplasms in regions of the Czech Republic in 19,504 males and 20,307 females in 1976–2005.

**Figure 7:**
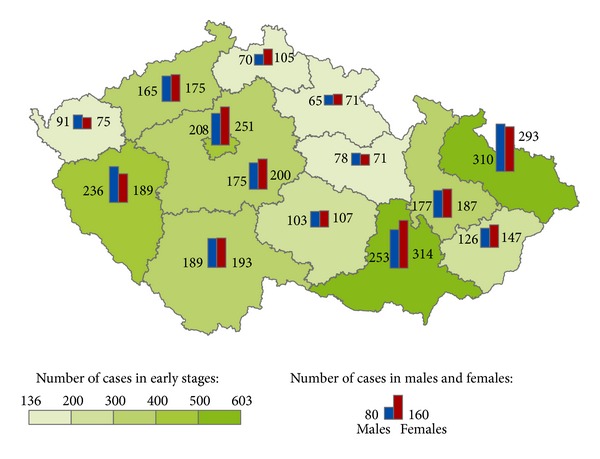
Early clinical stages of subsequent neoplasms after advanced clinical stages of primary neoplasms in regions of the Czech Republic in 2,246 males and 2,378 females in 1976–2005.

**Figure 8:**
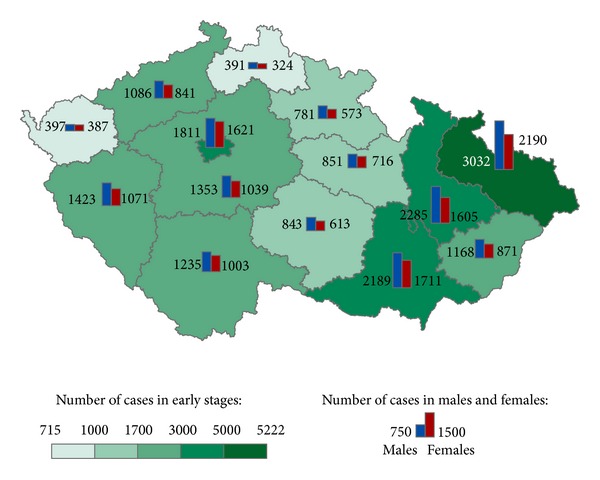
Early clinical stages of subsequent neoplasms after unknown clinical stages of primary neoplasms in regions of the Czech Republic in 18,845 males and 14,565 females in 1976–2005.

**Figure 9:**
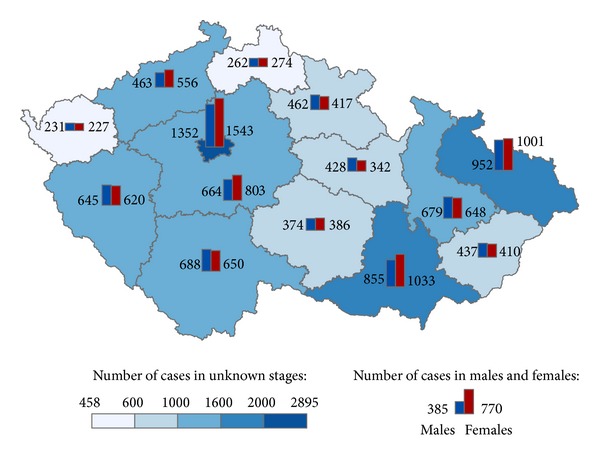
Unknown clinical stages of subsequent neoplasms after early clinical stages of primary neoplasms in regions of the Czech Republic in 8,492 males and 8,910 females in 1976–2005.

**Figure 10:**
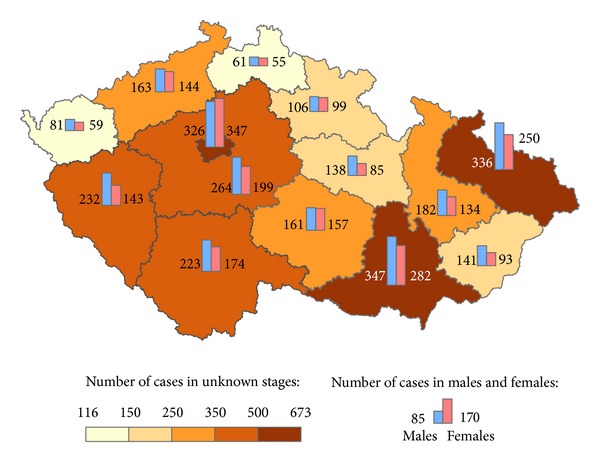
Unknown clinical stages of subsequent neoplasms after advanced clinical stages of primary neoplasms in regions of the Czech Republic in 2,761 males and 2,221 females in 1976–2005.

**Figure 11:**
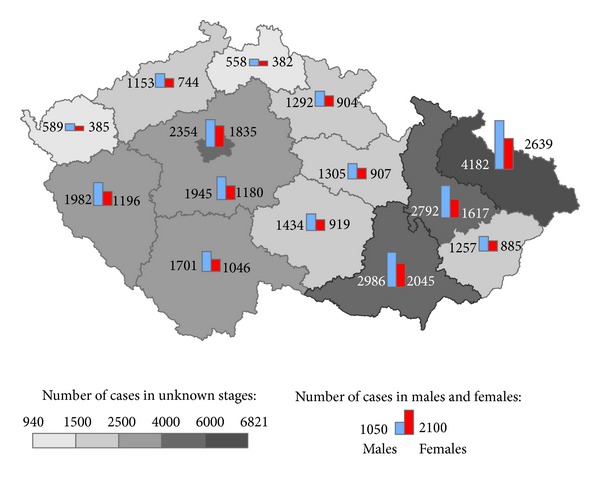
Unknown clinical stages of subsequent neoplasms after unknown clinical stages of primary neoplasms in regions of the Czech Republic in 25,530 males and 16,684 females in 1976–2005.

The comparison with total advanced stages in subsequent cancers was higher in regions nos. 11 (12.7%), 14 (12.1%), 4 (9.7%), and 1 (9.5%), while the comparison with all subsequent cancers in the relevant region was higher in regions nos. 6 (15.4%), 7 (14.7%), 4 (13.6%), 5 (13.6%) and 13 (12.4%) as an indicator of late diagnosis of subsequent cancer during medical surveillance (Table 3). Of the various comparisons are presented 9,037 advanced stages of subsequent cancers following primary neoplasms with higher value of 1603 (35.7%) gastrointestinal tract in men and 1718 (37.8%) in women, 1489 (33.2%) respiratory tract in men and 636 (14%) in women, 843 (18.5%) breast, 742 (16.3%), female genital organs, 498 (11.1%) male female genital organs, and 376 (8.4%) urinary tract in men and 218 (4.8%) in women (Figure 2).

Up to October 17, 2007, there were registered of 65,292 primary cancers of men 18,887 (28.9%) surviving and 46,405 (71.1%) deaths, of 59,970 primary cancers of women 22,274 (37.1%) surviving and 37,696 (62.9%) deaths. Of total surviving cases were 54.7% early stages in men and 58% in women, 5% advanced stages in men and 5.2% in women; the unknown stages featured 40.3% in men and 36.9% in women. Of total deceased cases were 30% early stages in men and 39.8% in women, 11% advanced stages in men and 12.1% in women; the unknown stages featured 11% in men and 12.1% in women.

## 4. Discussion

From ongoing analysis of Cancer Registry database were published the results of the MMNs of breast [7], skin [8], prostate [9], gastrointestinal tract [10], brain [11], lung [12], head and neck [13], testis [14], and penis cancer [15]. We pointed to the current and future relationships of MMNs with ethical and economic burden of the Czech population [16]. We used experiences of more than 50 references, concerning the MMNs. Unfortunately the excellent source which analysed the MMNs of SEER database [17] did not contain data about clinical stages as well as the most of another recent reference [18].

Registered new cases of malignant neoplasms contain also other primary subsequent cancer diagnoses, first described by Billroth and von Winiwarer in 1889 [19]. As their possible causes were assessed the previous radiotherapy [20–23] and chemotherapy [24–28], dialysis [29], transplantation [30–32], and genetic predisposition [33, 34]. Their relationship for the risk of the MMNs was not statistically significant. Nevertheless, there is an agreement that the mutual coincidence of these causes can promote the occurrence of subsequent cancers with high burden on patients, their families, and oncologists, including the extraordinary difficulty of palliative care in the terminal period. 

It can be assumed that the dispensary care can bring except the metastasis also other topographically and histologically different cancers. Their treatment has similar conditions by clinical stages even when the subsequent cancer can change the treatment scheme of the primary disease. 22,607 advanced stages, that is, 13.7% of all 165,050 subsequent cases during 29 years—is it high or low number? It is important to note that the number of subsequent advanced stages increased with advanced stage of primary disease. These results can contribute to the algorithms of dispensary care and early detection of the subsequent neoplasms. During 1977–2005, nearly every fifth cancer disease of the Czech population was associated with the occurrence of histologically different neoplasms.

The presented maps of MMNs by clinical stages are an example of using spatial analysis in our epidemiological research. Spatial epidemiology is the study of the geographical variation in disease risk, incidence, or prevalence [35]. As a growing field of research, spatial epidemiology provides new insights into multiple cancers as it pertains also to the management of permanent medical surveillance in cancer patients [36] and prevention in the health population by modern visualization [37].

## 5. Conclusion

Over 29 years, nearly one in five cancers registered in the Czech population was associated with additional cancer. A total of 165,050 subsequent neoplasms 13.7% were diagnosed in the advanced stage. The results suggest that information about multiple neoplasms and their clinical stages is necessary as a part of annual statistical cancer report. The most of subsequent advanced stages can be prevented by the therapeutic guidelines. 

## Figures and Tables

**Table 1:** New cancers and multiple malignant cases based on the Czech Cancer Registry 1976–2005 (up to October 17, 2007, data in 1976 from May).

Malignant neoplasm	ICD-10	Men				Women			
		New MN	% (a)	MMNs	% (b)	New MN	% (a)	MMNs	% (b)
Oral cavity, pharynx	C00-14	20673	2,8	2070	3,2	6241	0,9	540	0,9
Gastrointestinal tract	C15-26	193547	25,6	8862	13,6	162077	22,2	5882	9,8
Respiratory tract	C30-39	162315	21,4	5093	7,7	32263	4,4	887	1,5
Bone, articular cartilage	C40-41	1765	0,2	70	0,1	1320	0,2	50	0,1
Melanoma of skin	C43	14331	1,9	1626	2,5	15327	2,1	1518	2,5
Skin	C44	134727	17,8	30012	46	129787	17,8	23604	39,4
Mesothelial, soft tissue	C45-49	5489	0,7	321	0,5	5177	0,7	267	0,4
Breast	C50	978	0,1	128	0,2	118546	16,2	10352	17,3
Female genital organs	C51-58	0	0	0	0	115912	15,9	8785	14,7
Male genital organs	C60-63	74560	9,8	5265	8,1	0	0	0	0
Urinary tract	C64-68	67530	8,9	6278	9,6	32807	4,5	2221	3,7
Eye, brain, nervous system	C69-72	10734	1,4	305	0,5	9130	1,3	286	0,5
Thyroid and other glands	C73-75	3248	0,4	222	0,3	9357	1,3	585	1
Unspecified sites	C76-80	13539	1,8	392	0,6	13951	1,9	323	0,5
Lymphoid, haematol. tissue	C81-96	40071	5,3	2667	4,1	35056	4,8	1697	2,8

In situ	D00-09	7438	1	1161	1,8	35192	4,8	2301	3,8
Uncertain behaviour	D37-48	6449	0,9	820	1,3	7447	1	672	1,1

Total sites	C00-D48	757394	100	65292	100	729590	100	59970	100

% (a): relevant diagnosis in percent of all new cancers.

% (b): relevant diagnosis in percent of all malignant neoplasms.

**Table 2:** Primary and subsequent neoplasms by clinical stages 1976–2005 (Source: Czech Cancer Registry, up to October, 2007). Values of subsequent neoplasms in advanced stages are in bold.

Primary	Subsequent	Total	Men	Women
ST I	ST I	25936	13486	12450
	ST II	5220	2429	2791
	**ST III**	**2731**	1331	1400
	**ST IV**	**3441**	1893	1548
	Unknown	12389	6317	6072

Total		49717	25456	24261

ST II	ST I	5927	2534	3393
	ST II	2728	1055	1673
	**ST III**	**1310**	529	781
	**ST IV**	**1555**	735	820
	Unknown	5013	2175	2838

Total		16533	7028	9505

ST III	ST I	2163	953	1210
	ST II	980	389	591
	**ST III**	**951**	417	534
	**ST IV**	**954**	473	481
	Unknown	2842	1427	1415

Total		7890	3659	4231

ST IV	ST I	1040	644	396
	ST II	441	260	181
	**ST III**	**341**	191	150
	**ST IV**	**1014**	559	455
	Unknown	2140	1334	806

Total		4976	2988	1988

Unknown	ST I	26757	15184	11573
	ST II	6653	3661	2992
	**ST III**	**4324**	2483	1841
	**ST IV**	**5986**	3807	2179
	Unknown	42214	25530	16684

Total		85934	50665	35269

Total all sites		165050	89796	75254

**Table 3:** Advanced stages of subsequent neoplasms by regions 1976–2005 (Source: Czech Cancer Registry, up to October, 2007). Names of regions were changed to numbers because of anonymization.

Region	ADV-EAR	ADV-ADV	ADV-UNKN	Total	% (a)	% (b)
1	922	290	498	1710	**9,5**	8,8
2	781	295	455	1531	8,5	11,6
3	679	213	338	1230	6,8	10,6
4	818	357	564	1739	**9,7**	**13,6**
5	299	118	196	613	3,4	**13,6**
6	793	261	434	1488	8,3	**15,4**
7	375	112	186	673	3,7	**14,7**
8	284	106	232	622	3,5	8,5
9	321	99	217	637	3,5	8,7
10	391	180	309	880	4,9	11,3
11	1116	425	737	2278	**12,7**	11,8
12	675	186	409	1270	7,1	8
13	568	208	337	1113	6,2	**12,4**
14	1015	410	748	2173	**12,1**	9,6

ADV-EAR: advanced stages of subsequent cancers following early stages of primary cancers.

ADV-ADV: advanced stages of subsequent cancers following advanced stages of primary cancers.

ADV-UNKN: advanced stages of subsequent cancers following unknown stages of primary cancers.

% (a): percent of total advanced stages in subsequent cancers.

% (b): percent of subsequent cancers in the relevant region.
